# Physiological and Transcriptomic Analyses Reveal the Mechanisms of Compensatory Growth Ability for Early Rice after Low Temperature and Weak Light Stress

**DOI:** 10.3390/plants11192523

**Published:** 2022-09-26

**Authors:** Hui Wang, Lei Zhong, Xiaoquan Fu, Shiying Huang, Haihui Fu, Xiang Shi, Lifang Hu, Yicong Cai, Haohua He, Xiaorong Chen

**Affiliations:** 1 Key Laboratory of Crop Physiology, Ecology and Genetic Breeding, Ministry of Education, Jiangxi Agricultural University, Nanchang 330045, China; 2 Jiangxi Super Rice Engineering Technology Center, Jiangxi Agricultural University, Nanchang 330045, China; 3 College of Agriculture, Jiangxi Agricultural University, Nanchang 330045, China

**Keywords:** rice genotype, combined low temperature and weak light stress, compensatory growth, antioxidant enzymes, nitrogen metabolizing enzymes, endogenous hormones, transcriptome

## Abstract

“Late spring coldness” (T) is a frequent meteorological disaster in the spring in southern China, often causing severe yield losses of direct-seeded early rice. In this study, we investigated the mechanisms underlying the differences in the compensatory growth ability of different rice genotypes by focusing on agronomic traits, physiological indicators, and transcriptome. The results showed that there were significant differences in the compensatory growth recovery ability of different genotypes after a combination of four days of low temperature and weak light stress. Only the strong compensatory growth genotype B116 was able to grow rapidly and reduce soluble protein and H_2_O_2_ concentrations rapidly after stress. By analyzing enzyme activity as well as endogenous hormone concentration, we found that the high superoxide dismutase (SOD), peroxidase (POD), and catalase (CAT) activities and high levels of abscisic acid (ABA) could reduce the damage of B116 during stress. Meanwhile, higher glutamine synthetase (GS) and nitrate reductase (NR) activity and higher levels of gibberellin A_3_(GA_3_), indoleacetic acid (IAA), and zeatin nucleoside (ZR) could enable B116 to grow rapidly after stress. The identified differentially expressed genes (DEGs) indicated that there were large differences in POD-related genes and gibberellin metabolism between B116 and B144 after stress; RT-PCR quantification also showed a trend consistent with RNA-seq, which may be an important reason for the differences in compensatory growth ability.

## 1. Introduction

As a major food crop, rice (*Oryza sativa* L.) ranks first in China in terms of sown area and production [1]. In line with socio-economic development, there is a growing shortage of labor engaged in rice production [2]. Compared to traditional seedling raising and transplanting, direct-seeded rice is easy to manipulate mechanically, which can improve production efficiency and greatly reduce labor pressure [3,4]. The United States and developed countries in Europe have basically achieved fully mechanized direct seeding of rice, and the area of direct seeded rice in China and other Asian countries is also rising year by year [5]. However, low temperature stress, such as the “late spring coldness”, is a major factor limiting rice yields [6] which often occurs in southern China and the Yangtze River Basin of double-season rice cropping areas and causes serious damage to early rice production [7,8]. Transplanted rice can be insulated with plastic film during seedling growth, while direct-seeded early rice is often susceptible to stresses such as low temperature, weak light, and weed damage which result in slow growth, leaf discoloration, stunting, and even rotten or dead seedlings [9,10,11]. In 2008 and 2010, the seedling emergence rate of direct-seeded rice in southern China and the Yangtze River Basin was significantly reduced due to “late spring coldness”, which seriously affected early rice production [12,13]. In addition, due to global warming, the sowing date of early rice was advanced; however, the frequency of low-temperature events did not decrease significantly and the possibility of cold damage to direct-seeded early rice increased instead [14].

In terms of physiology, low temperature will lead to a large accumulation of reactive oxygen species (ROS) in rice plants. ROS can disrupt cell membrane integrity and cause severe physiological and biochemical damage by excessive oxidation of proteins, DNA, and other important macromolecules [15,16,17,18]; higher plants can scavenge ROS through antioxidant systems [19]. Moreover, low temperature stress leads to a decrease in photosynthesis-related enzyme activity, chlorophyll concentration, reduced light energy utilization, and growth stagnation in rice, resulting in inhibited photosynthetic feedback [20,21]. The combined stresses of low temperature and high humidity reduce the activity of various metabolic and antioxidant enzymes in rice seedlings, while also reducing rice growth performance by blocking photosynthetic respiratory reactions and regulating endogenous hormones [22]. The low temperatures that occur during the “late spring coldness” are often accompanied by weak light stress. The shading treatment of 61 rice varieties (lines) at the seedling stage showed that the root length, leaf age, root volume, and root dry weight of most varieties decreased by varying degrees [23]. The biomass and leaf photosynthesis rate (*P_n_*) both significantly decreased under weak light stress, while the leaf *P_n_* in super hybrid rice was higher than that in conventional rice [24]. Meanwhile, weak light stress affects the activities of key enzymes related to nitrogen metabolism, such as glutamine synthetase (GS), glutamate-oxaloacetate transaminase (GOT), and glutamate-pyruvate transaminase (GPT) [25]. Currently, most studies have focused on exploring how the effects of a combination of low temperature and weak light stresses affect rice growth or have investigated the mechanism of rice seedling damage from various perspectives, such as physiological indicators, transcriptomics, and proteomics [26,27]; however, less attention has been given to the compensatory growth of rice seedlings after stress. Investigating the mechanisms related to the rapid growth ability for different rice genotypes after stress is beneficial to the improvement of rice breeding and cultivation measures. Meanwhile, researchers analyzed the temperature data of 87 meteorological stations in Jiangxi Province in China (from 1961 to 2018) and found that the frequency of severe “late spring coldness” disasters in Jiangxi Province in China gradually decreased over the past 30 years, while the frequency of mild and moderate “late spring coldness” increased significantly [28]. Therefore, direct-seeded early rice in southern China and the Yangtze River Basin, used as double-season rice areas in China, mainly face problems involving the slow and weak growth of seedlings caused by sub-low temperature damage, associated with seasonal changes such as mild and moderate “late spring coldness”. The competitive advantage of weeds is stronger than that of rice seedlings under low temperature, which can easily cause too many weeds and make it difficult for rice seedlings to grow normally; it is inconvenient to apply herbicides because of the weak growth of rice seedlings. Therefore, direct-seeded early rice is often accompanied by serious weed damage after the “late spring coldness” [29]. On the other hand, most plants usually have a transient and rapid compensatory growth after experiencing short-term stress during growth, and appropriate mild stress helps plants to better adapt to the external environment [30,31,32]. In ecology, it is usually called the growth compensation effect or rebound effect, and there have been numerous studies on crops that have investigated rewatering after drought and nutrient deficiency and compensation [33,34,35].

Accordingly, we believe that rice seedlings should have a period of compensatory growth when temperature and light have returned to normal after a brief period of combined low temperature and weak light stresses, similar to what is observed with “late spring coldness”. This will result in morphological and physiological changes in rice seedlings, and there will be differences in compensatory growth ability among different genotypes of rice. In fact, we found great variability in compensatory growth ability among different rice varieties (lines) after combined low temperature and weak light stresses in our preliminary screening experiments. Among these variations, the height ratio (treated plant height divided by control plant height) of the strong compensatory growth genotype B116 was 0.595 at day 0 (the end of the treatment day) and changed to 0.839 after 6 days, while the height ratios of the weak compensatory growth genotypes B144 and B811 were both 0.561 at day 0 and changed to 0.629 and 0.613 after 6 days, respectively. As mentioned earlier, researchers have found that crops experiencing low temperature stress undergo dramatic physiological changes, particularly in relation to enzyme activity, endogenous hormone levels, and the expression of related genes. However, there are few reports on the mechanisms related to the differences between strong and weak compensatory growth genotypes after combined low temperature and low-light stress in rice. Therefore, we speculate that the strong compensatory growth genotype should have greater advantages in nitrogen metabolism, antioxidant enzyme activity, and growth-promoting hormone levels after low temperature and weak light stress. To further test the above hypothesis, we conducted this experiment in an artificial climate chamber, and three materials with significant differences for compensatory growth after stress were selected to investigate the growth of direct-seeded rice seedlings after stress. The morphological indicators, physiological indicators, and transcriptomic data were analyzed to provide insight into the effects of stress on rice growth and the mechanisms related to the differences in compensatory growth among genotypes after stress. This study also has general relevance for crop response to non-lethal low-temperature stress and compensatory growth after stress.

## 2. Materials and Methods

### 2.1. Plant Materials and Growth Conditions

In our preliminary experiment, 131 early rice materials were subjected to low temperature and weak light stress, and they were classified into strong compensatory growth type, medium compensatory growth type, and weak compensatory growth type according to differences in compensatory growth ability. We also selected B116 (R310/R974, F_17_; strong compensatory growth type), B144 (ZaoxianST66-3, weak compensatory growth type), and B811 (Zhonghui286, weak compensatory growth type) for the barrel planting trials. The experiment was conducted from June to July of 2021 in an artificial climate chamber at the Key Laboratory of Crop Physiology, Ecology, and Genetic Breeding, Ministry of Education, Jiangxi Agricultural University, Nanchang, Jiangxi Province, China (115°50′ E, 28°46′ N). The plastic bucket used had an upper internal diameter of 29.0 cm, a bottom internal diameter of 23.5 cm, and a height of 24.0 cm. The soil used in the experiment was taken from the shallow layer (0~20 cm) of the rice field and was then placed in the courtyard of the crop genetic breeding experiment base at Jiangxi Agricultural University to dry naturally, before then being crushed with an FT-1000A soil crusher (Ambo Instruments Co., Ltd., Shandong, China). Each bucket was filled with 10 kg of air-dried soil and 4 g of compound fertilizer (pure nitrogen-P_2_O_5_-K_2_O = 15%-15%-15%) as substrate, and the soil used was soaked in water for 5 days before the start of the experiment.

### 2.2. Experimental Design

The rice seeds were disinfected with a 3% sodium hypochlorite solution (20 min) before the start of the experiment, rinsed three times with water, placed in germination bags, soaked in water (30 °C) until they showed white, and then sown directly in plastic buckets on 17 June. Treatment was started when rice seedlings grew to the 2-leaf to 3-leaf stage (25 June). The test conditions are based on the national standard of the People’s Republic of China for inversion weather indicators and “late spring coldness” in Jiangxi Province [28,36]. The control group (CK) was set to a 12-h photoperiod (6:00~18:00) with light intensity of 533 μmolm^−2^s^−1^, the daytime (6:00~18:00) and nighttime (18:00~6:00) temperatures were 27 °C and 25 °C, respectively, and the relative humidity was set to 75%. For T, the photoperiod was set to 12 h (6:00~18:00) with reference to the “late spring coldness” standard and the average daily temperature was set to 12 °C, that is, the daytime (6:00~18:00) and nighttime (18:00~6:00) temperature was 14 °C and 10 °C, respectively. Light intensity was set to 267 μmolm^−2^s^−1^ (50% that of the CK) and relative humidity was set to 75% for four days. On the last day of the low-temperature and low-light treatment, we performed the first sampling and recorded it as day 0. After the sampling we set the temperature and light for the treatment group to be the same as the control group and performed subsequent sampling surveys (day 3, day 6, day 9, and day 12). We flash-froze the collected samples in liquid nitrogen and stored them in an ultra-low temperature refrigerator (−80 °C) in order to be able to measure physiological and biochemical parameters.

### 2.3. Agronomic Trait Determination

Starting from the last day of treatment (day 0), data was collected every 3 days for a total of 5 measurements. Whole seedlings with uniform growth were selected to measure plant height and were then washed and placed in an oven at 80 °C for drying before their dry weight was measured. Five biological replicates were performed per treatment.

### 2.4. Determination of Soluble Protein, H_2_O_2_ Concentration, and Antioxidant Enzyme Activity

Soluble protein and H_2_O_2_ concentration and antioxidant enzyme activity were measured continuously at day 0, day 1, day 3, day 6, and day 12 after the treatment. For each treatment, uniformly growing seedlings were selected and the leaf tissues were taken as samples. They were then washed and immediately frozen in liquid nitrogen and stored at −80 °C in a refrigerator. A total of three biological replicates were performed. The above indexes were measured using a Bradford Protein Assay Kit from Beyotime Biotechnology Co., Ltd. (Shanghai, China) and a superoxide dismutase (SOD) assay kit, catalase (CAT) assay kit, and peroxidase assay kit from Nanjing Jiancheng Bioengineering Institute, respectively.

### 2.5. Determination of NR, GS, and Rubisco Activities

NR, GS, and Rubisco activity was measured continuously at day 0, day 6, and day 12 after the treatment. For each treatment, uniformly growing seedlings were selected and the leaf tissues were taken as samples. They were then washed and immediately frozen in liquid nitrogen and stored at −80 °C in a refrigerator. A total of three biological replicates were performed. The assays were performed using an NR-2-W, GS-2-Y, and RUBPS-2A-Y commercial kit, respectively (Comin Biotechnology Co. Ltd., Suzhou, China).

### 2.6. Determination of Endogenous Hormone Concentrations

The endogenous hormone concentrations of GA_3_, ABA, IAA, and ZR in the leaves of the above samples were determined by high performance liquid chromatography according to the instructions of a commercial kit (Comin Biotechnology Co., Ltd., Suzhou, China).

### 2.7. RNA Extraction and Transcriptome Sequencing

Total RNA was extracted from seedling leaves at day 3 after stress by using a Trizol reagent kit (Invitrogen, Carlsbad, CA, USA). RNA quality was assessed on an Agilent 2100 Bioanalyzer (Agilent Technologies, Palo Alto, CA, USA). After total RNA was extracted, mRNA was enriched by Oligo (dT) beads. The enriched mRNA was then fragmented into short fragments using fragmentation buffer and was reverse transcribed into cDNA with random primers. The cDNA fragments were then purified with a QiaQuick PCR extraction kit (Qiagen, Venlo, The Netherlands), the ends were repaired, poly (A) was added, and the fragments were then ligated to Illumina sequencing adapters. After PCR amplification, sequencing was performed using an Illumina HiSeq2500 from Gene Denovo Biotechnology Co (Guangzhou, China).

### 2.8. Quantitative RT-PCR Validations

To verify the reliability of the transcriptome results, 8 DEGs co-expressed in B116-CK, B116-T, B144-CK, and B144-T were selected, and the primers were designed using SnapGene 3.2.1 (the sequences are listed in Supplementary Table S1) and were synthesized by Tsingke Biotechnology Co., Ltd. (Beijing, China) before then being synthesized using the ChamQ Universal SYBR^®^ qPCR Master Mix Q711 (Vazyme Biotech Co., Ltd., Nanjing, China). The RNA used for quantitative RT-PCR was the same as that used to construct the cDNA library, and the related genes were normalized to Actin transcript levels by the 2^−ΔΔCt^ method [37].

### 2.9. Statistical Analysis

Data obtained were analyzed for significance of differences and plotted with SPSS 26.0 and GraphPad Prism 8.0. The data were analyzed through analysis of variance and the differences between treatments were tested using the least significant difference (LSD) test with a probability level of 0.05.

GR = (I_n+3_ − I_n_)/I_n_

GR, growth rate; I, agronomic trait indexes; n, the number of days of growth after the end of stress.

## 3. Results

### 3.1. Differences in Agronomic Traits

**Figure 1:**
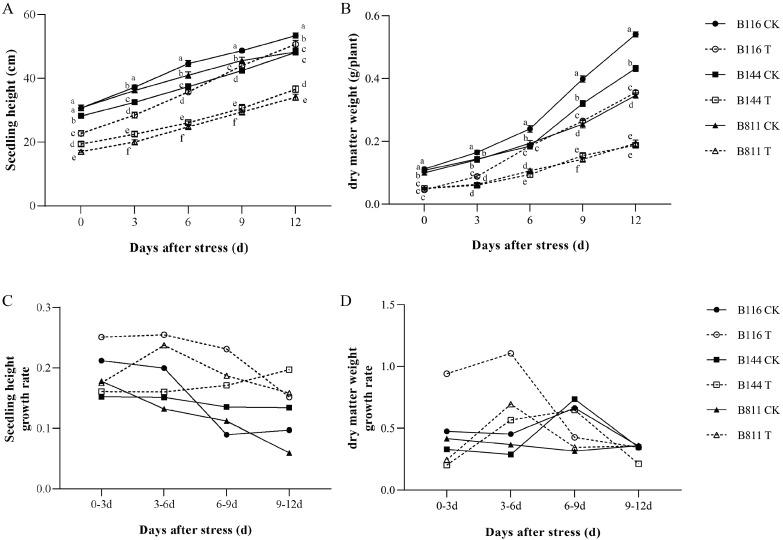
Changes in seedling height, dry matter weight (**A**,**B**) and seedling growth rate and dry matter weight growth rate (**C**,**D**) of direct-seeded early rice seedlings for the three materials after a combination of low temperature and weak light stress. CK, control; T, low temperature and weak light stress. Error bars represent standard deviation (*n* = 15). Data are mean ± SD. Different lowercase letters indicate significant differences at *p* < 0.05 based on Duncan’s test.

To investigate the differences in compensatory growth ability for B116, B144, and B811 after stress, the seedling height and dry matter weight of each material at day 0, day 3, day 6, day 9, and day 12 after the end of stress were measured. As shown in Figure 1A,B, B116 in the CK had the highest seedling height and dry matter weight at day 0, while seedling height and dry matter weight were the lowest with B144 and B811, respectively. Compared to the CK, the seedling height and dry matter weight of each material decreased significantly on the day after stress in T. On day 0, there was a significant difference in plant height among the materials. At the stage of suitable temperature and light, the growth rate of B116 was significantly faster than that of B144 and B811. The difference in plant height gradually became larger alongside increasing growth time in the suitable environment. For the index of dry matter weight, that of B116 was lower than the other two materials at the end of the stress period and reached a significantly different level from that of B144. However, the rate of dry matter accumulation for B116 was significantly higher compared to the other two materials at the compensatory growth stage. It can be seen that the stress treatment amplified the difference between B116 and the other two genotypes, both in seedling height and dry matter weight, indicating that B116 has strong compensatory growth performance and its plant height can even be close to the CK. From the growth rate of seedling height and dry matter weight (Figure 1C,D), it can be seen that the growth rate of B116 in the CK was slightly higher than that of B144 and B811 on day 0 to day 6, but did not show large differences, while the growth rate of B116 in T was much higher than that of B144, B811, and B116 in the CK on day 0 to day 6.

### 3.2. Differences in Soluble Protein, H_2_O_2_ Concentration, and Antioxidant Enzyme Activity

**Figure 2:**
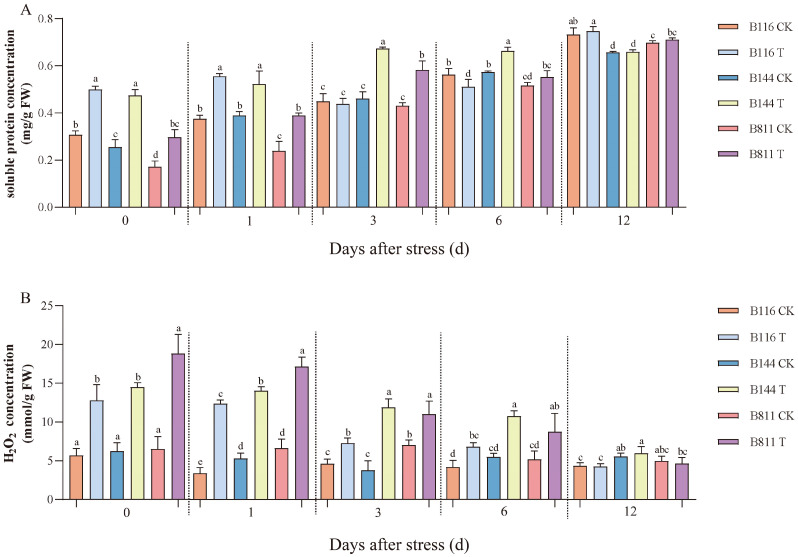
Changes in soluble protein (**A**) and H_2_O_2_ (**B**) concentration for direct-seeded early rice seedlings after a combination of low temperature and weak light stress. CK, control; T, low temperature and weak light stress. Error bars represent standard deviation (*n* = 3). Data are mean ± SD. Different lowercase letters indicate significant differences at *p* < 0.05 based on Duncan’s test.

**Figure 3:**
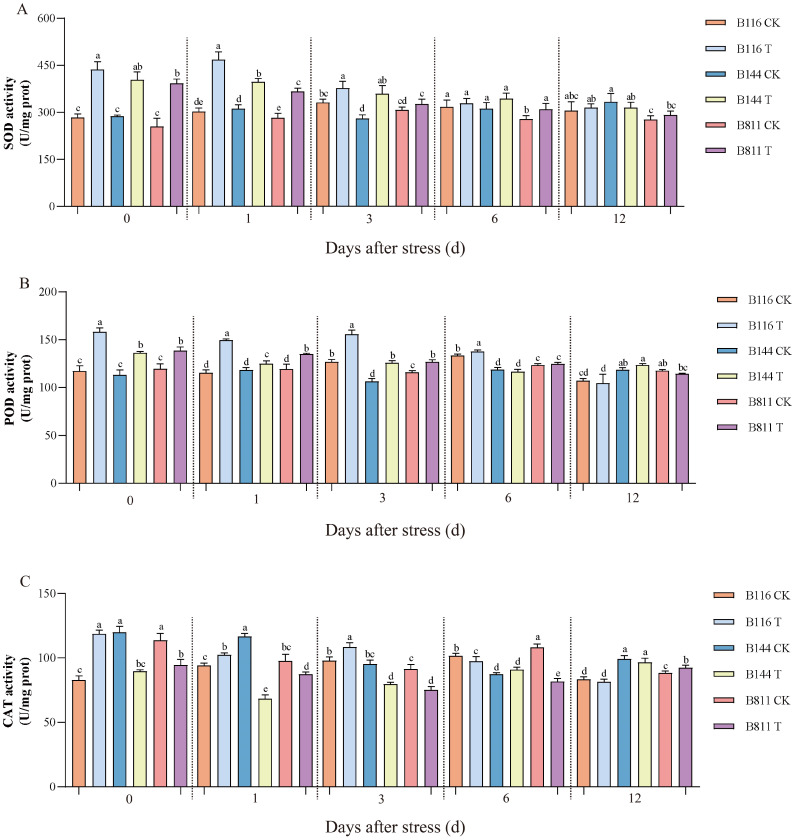
Changes in superoxide dismutase (**A**), peroxidase (**B**), and catalase (**C**) activities for direct-seeded early rice seedlings after a combination of low temperature and weak light stress. CK, control; T, low temperature and weak light stress. Error bars represent standard deviation (*n* = 3). Data are mean ± SD. Different lowercase letters indicate significant differences at *p* < 0.05 based on Duncan’s test.

Low temperature and weak light stress had significant effects on soluble protein and H_2_O_2_ concentrations (Figure 2). At day 0, the soluble protein and H_2_O_2_ concentrations of all three rice materials increased substantially. After three days, the soluble protein and H_2_O_2_ concentrations in B116 gradually decreased and approached the level of the CK. B144 and B811 also recovered somewhat after stress, but these two indicators were still significantly higher than the level of the CK, especially the H_2_O_2_ concentration. The activity of antioxidant enzymes is important for reducing the damage to rice caused by low temperature and weak light stress. On day 0, superoxide dismutase (SOD), peroxidase (POD), and catalase (CAT) activities were significantly higher in B116 compared to the CK (Figure 3) and remained at a high level from day 0 to day 6. In contrast, compared to the CK, POD activity for B144 and B811 only showed a small increase, and CAT activity even decreased. On the contrary, compared to the CK, SOD and POD activities for B144 and B811 only showed a small increase, and CAT activity even showed a decrease, and their activities were lower than those of B116 from day 0 to day 6.

### 3.3. Differences in NR, GS, and Rubisco Activity

NR is the key enzyme for the transformation of nitrate nitrogen into ammonia nitrogen in plants. GS is an enzyme within organisms that uses other substances to synthesize glutamine and it is an important physiological index to measure the level of nitrogen assimilation in plants. Rubisco is an important regulator of carbon metabolism in photosynthesis, and it catalyzes the first major carbon fixation reaction in the Calvin cycle of photosynthesis.

**Figure 4:**
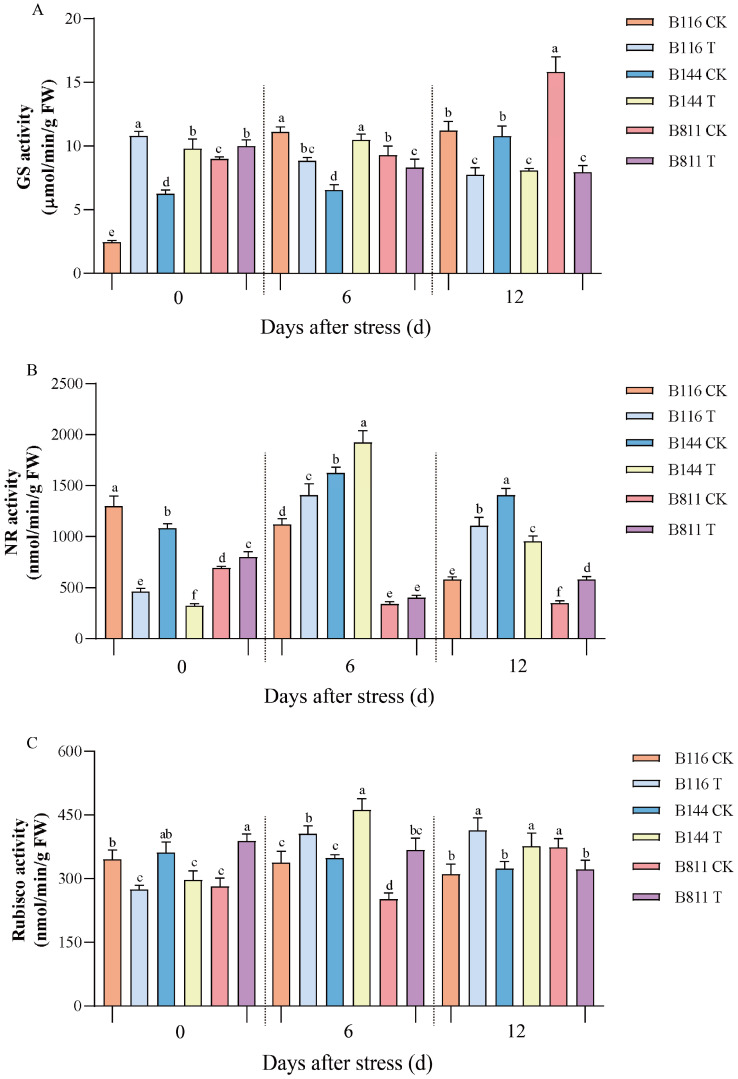
Changes in glutamine synthetase (GS) (**A**), nitrate reductase (NR) (**B**), and ribulose bisphosphate carboxylase oxygenase (Rubisco) (**C**) activity for direct-seeded early rice seedlings after a combination of low temperature and weak light stress. Different lowercase letters indicate significant differences at *p* < 0.05. CK, control; T, low temperature and weak light stress. Error bars represent standard deviation (*n* = 3). Data are mean ± SD. Different lowercase letters indicate significant differences at *p* < 0.05 based on Duncan’s test.

As shown in Figure 4A, the activity of GS for all materials in T increased to different degrees on day 0 compared to the CK, and that of B116 showed the largest increase of 342.6%, while B811 the smallest increase of 11.3%. The activity of GS for B116 and B811 gradually decreased with the increase in compensatory growth time, and B144 reached the highest value on day 6 and then slowly decreased. At day 12, B811 in T showed a 49.7% decrease compared to that in the CK. NR activity for B116 and B144 in T (Figure 4B) showed an increasing and then decreasing trend, while B811 was diametrically opposite. NR activity for B116 and B144 in T showed a 64.5% and 70.1% decrease at day 0, respectively, compared to the CK, while showing an increase at day 6 which was 25.9% and 18.3% higher than the CK, respectively. After 12 days of compensatory growth, the NR activity of B116 was significantly higher than that of B144 (90.0% higher compared to the CK), while B144 showed a 32.3% decrease compared to the CK. The NR activity of B811 in T was 15.1%, 28.0% and 67.5% higher than that of the CK on day 0, day 6, and day 12, respectively; however, the overall activity level was low. The levels and trends of GS and NR activity for B116 and B144 after stress were relatively similar. However, the elevation of GS and NR activities in B116 T compared to B116 CK was significantly greater than that of B144 T compared to B144 CK. As shown in Figure 4C, B116 and B144 showed a decrease in Rubisco activity compared to the CK on day 0, while B811 showed an increase of 37.9%. On day 6, the Rubisco activity for all three materials in T showed a large increase compared to the CK, with the difference being that B116 and B144 showed an increasing trend and only B811 showed a decreasing trend. With the extension of compensatory growth time, the Rubisco activity of B116 remained stable, while that of B144 and B811 showed a decreasing trend.

### 3.4. Differences in GA_3_, ABA, IAA, and ZR Concentrations

**Figure 5:**
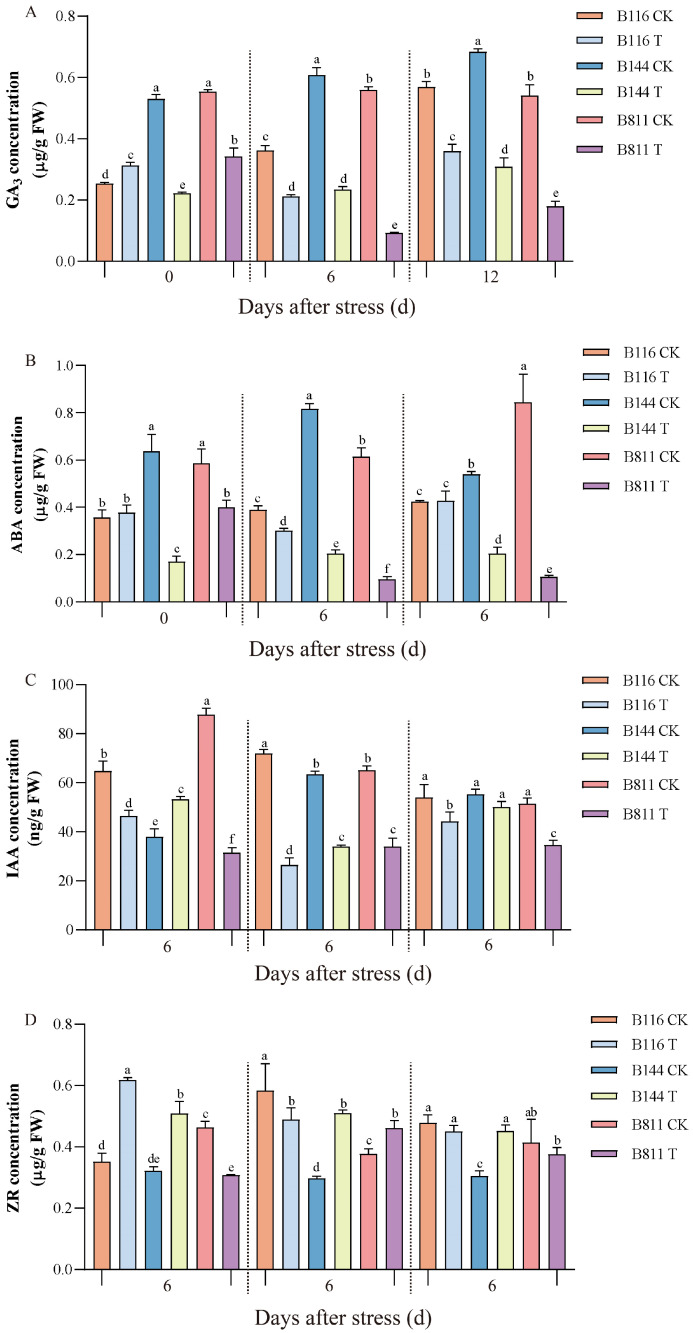
Changes in gibberellin A_3_ (GA_3_) (**A**), abscisic acid (ABA) (**B**), indoleacetic acid (IAA) (**C**), and zeatin riboside (ZR) (**D**) concentrations for direct-seeded early rice seedlings after a combination of low temperature and weak light stress. Different lowercase letters indicate significant differences at *p* < 0.05. CK, control; T, low temperature and weak light stress. Error bars represent standard deviation (*n* = 3). Data are mean ± SD. Different lowercase letters indicate significant differences at *p* < 0.05 based on Duncan’s test.

Endogenous plant hormones such as GA, ABA, IAA, and ZR play an important role in the growth regulation of rice. Under stress, rice can enhance its resistance to stress by regulating the ABA/GA_3_ balance. After the restoration of suitable temperature and light, GA_3_ can affect rice plant height by activating cell division in meristematic tissue and promoting cell elongation [38,39]. IAA and CTK act synergistically in a variety of cellular and physiological processes, such as cell expansion and apical dominance [40,41]. From the end of stress, we tracked the changes in GA_3_, ABA, IAA, and ZR concentrations. On day 0, GA_3_ concentrations decreased by 58.5% and 38.2% in B144 and B811, respectively, compared to the CK and remained much lower than the CK until day 12 (Figure 5A). This difference reached its maximum at day 6 (62.3% and 83.9% decrease, respectively). In contrast, B116 showed a small increase in GA_3_ concentration of 24.0% on day 0 compared to the CK and was always lower than the CK afterwards; however, its decline was less than that of B144 and B811. It should be noted that the changes in GA_3_ concentrations for B116 and B811 in T were exactly opposite to those in the CK. The GA_3_ concentration of B116 in the CK was significantly lower than that of B144 and B811 from day 0 to day 6. However, the GA_3_ concentrations of B116 in T were at a relatively high level from day 0 to day 12. In the CK, B811 had a high level of GA_3_ from day 0 to day 12; however, in T, the GA_3_ concentrations were significantly lower than those of B116 and B144 from day 6 to day 12. The ABA concentrations of B116 in T did not show any major changes on day 0 compared to the CK (Figure 5B), while the concentration of B144 and B811 decreased by 73.4% and 32.2%, respectively, and the ABA concentrations of B144 were significantly lower than those of B116 and B811. The ABA concentrations of B116 and B144 in T fluctuated little during the subsequent stages, and the ABA concentration of B116 decreased slightly on day 6 before then gradually increasing to match that of the CK; there was no significant change in B144. The ABA concentration of B811 was at a high level on day 0 but decreased rapidly during the subsequent stage and was significantly lower than that of B116 and B144 from day 6 to day 12. The IAA trend of B116 in T was similar to that of GA_3_ and ABA and was significantly higher than the remaining two materials at day 0 and day 12, while only significantly higher than that of B144 at day 6 (Figure 5C). The ZR concentration of B116 in T gradually decreased after day 0 and was significantly higher than the remaining two materials at day 0, while only significantly higher than B811 at day 12 (Figure 5D).

### 3.5. Transcriptome Sequencing and Functional Identification of B116 and B144

**Figure 6:**
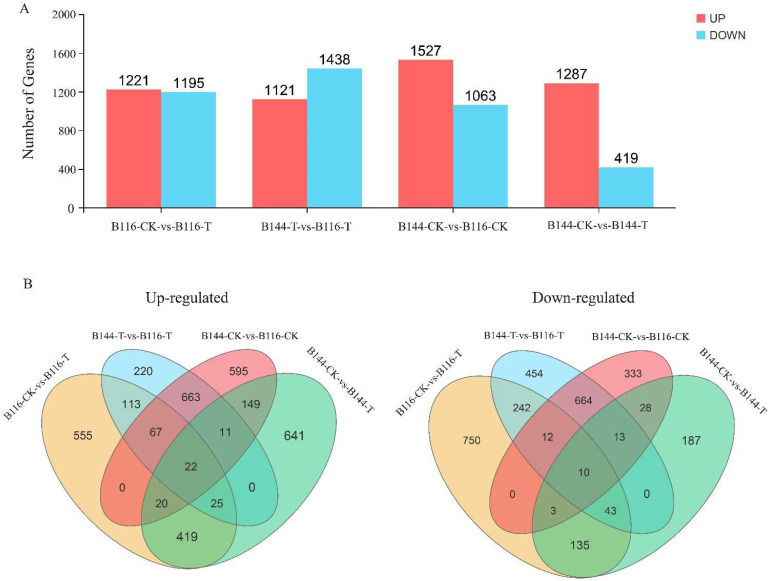
Comparison of gene expression differences between early rice materials B116 and B144 with and without a combination of low temperature and weak light stress. (**A**) The number of differentially expressed genes (DEGs) for the four pairwise comparison groups is indicated. (**B**) The Venn diagram represents the number of DEGs in different comparison groups, where the overlapping part of the graph indicates the common DEG between different comparison groups.

Transcriptome analysis can help us reveal the differences in compensatory growth after stress among different rice materials at the molecular level. Here, we subjected B116-CK, B116-T, B144-CK, and B144-T samples from day 3 after stress to transcriptome analysis (RNA-seq), and each sample yielded an average of 47 million clean reads (range 39 million to 60 million) with a Q20 ratio of 97.4% (range 96.2% to 97.9%), a Q30 ratio of 93% (range 90.4% to 94.2%), and a GC content percentage of 53.75% (range 52.76% to 54.11%). In this study, we designed four comparison groups: B116-CK vs. B116-T, B144-CK vs. B144-T, B144-CK vs. B116-CK, and B144-T vs. B116-T. We then performed differentially expressed gene (DEG) analysis (FDR < 0.05, |log2(FC)| > 1) (Figure 6A). A total of 2416 DEGs (1221 up-regulated and 1195 down-regulated) were identified in B116-CK vs. B116-T, 1706 DEGs (1287 up-regulated and 419 down-regulated) were identified in B144-CK vs. B144-T, and 2559 DEGs (1121 up-regulated and 1438 down-regulated) were identified in B144-T vs. B116-T. From the Venn diagram (Figure 6B), it can be seen that 677 genes were universally regulated in B116-CK vs. B116-T and B144-CK vs. B144-T at day 3 after stress, including 486 up-regulated genes and 191 down-regulated genes, while a total of 22 up-regulated genes and 10 down-regulated genes were universally regulated in the four groups.

**Figure 7:**
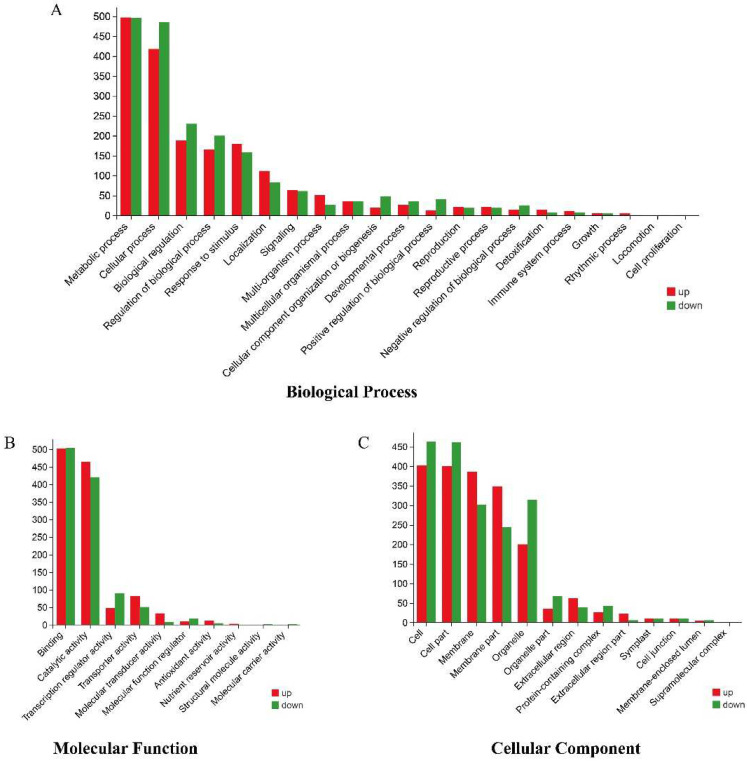
GO enrichment analysis of DEGs in the B116-CK vs. B116-T comparison group, where the *X*-axis represents different GO terms and the *Y*-axis represents the number of DEGs. (**A**) Biological process, (**B**) molecular function, (**C**) cellular component.

**Figure 8:**
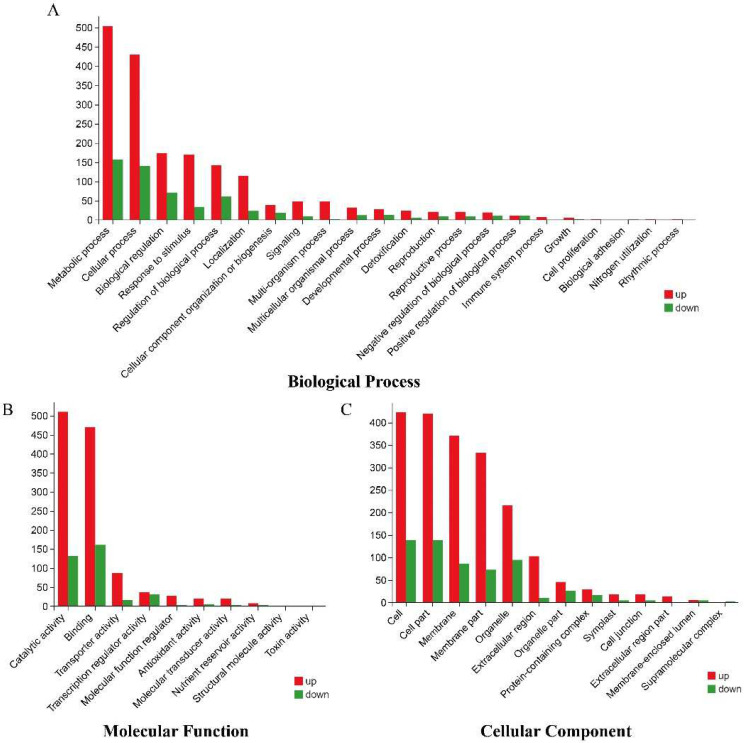
GO enrichment analysis of DEGs in the B144-CK vs. B144-T comparison group, where the *X*-axis represents different GO terms and the *Y*-axis represents the number of DEGs. (**A**) Biological process, (**B**) molecular function, (**C**) cellular component.

GO analysis of the DEGs (Figure 7 and Figure 8, Supplementary Figures S1 and S2) showed that there were 54 enriched GO terms in the three categories of biological processes, cellular components, and molecular functions, and the enrichment types were relatively consistent across comparison groups; however, the number of enriched genes differed between comparison groups.

The results showed that the metabolic process (GO:0008152) and cellular process (GO:0009987) were significantly enriched in the biological process category; cell (GO:0005623), cell part (GO:0044464), and membrane (GO:0016020) were significantly enriched in the cellular component category; and binding (GO:0005488) and catalytic activity (GO:0003824) were the most dominant GO terms in the molecular function category (Supplementary Tables S2–S5).

**Figure 9:**
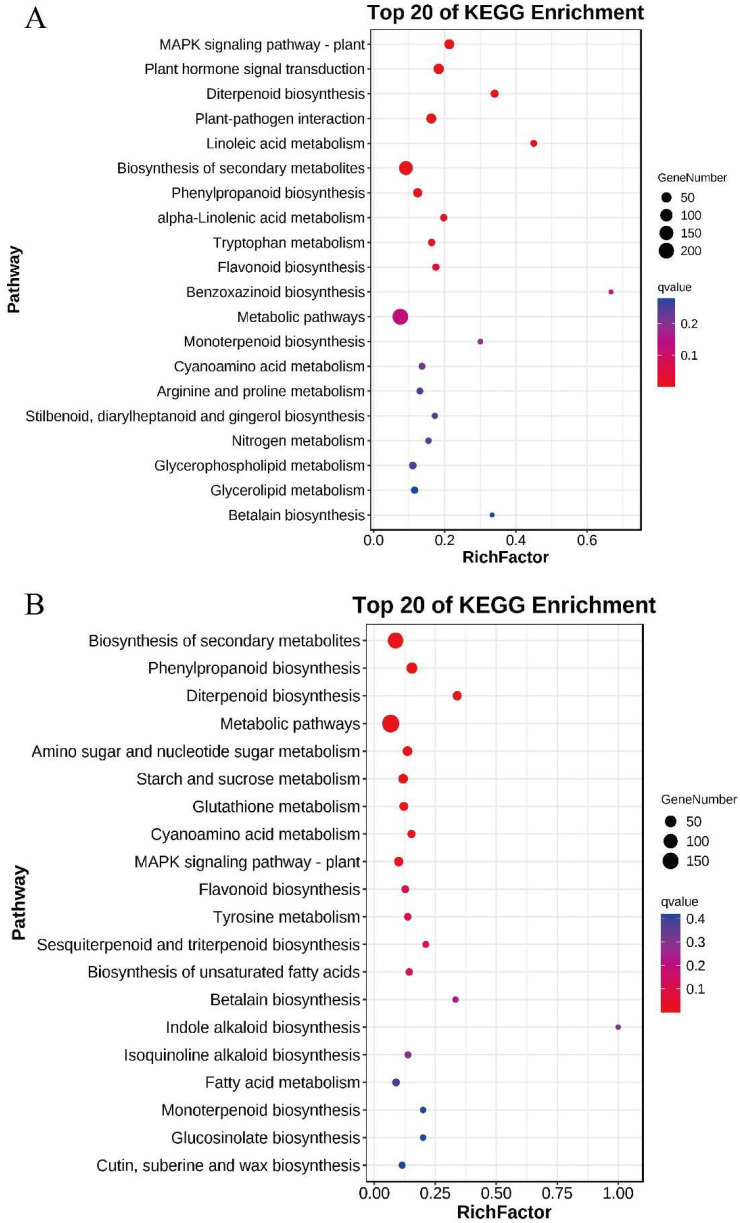
Top 20 of KEGG pathway enrichment analysis of the DEGs. (**A**,**B**), represent B116-CK vs B116-T and B144-CK vs B144-T respectively. The *X*-axis indicates the rich factor (Divide the number of differential genes in the pathway by all the numbers in the pathway) and the *Y*-axis indicates the KEGG pathway. Circle size indicates the number of DEGs in the pathway and the colors indicate Q value of DEGs.

The KEGG results show the main enrichment pathways in each comparison group (Figure 9 and Supplementary Figure S3). A total of 388 DEGs were identified in the B116-CK vs. B116-T comparison group, and their main enrichment pathways were “Plant hormone signal transduction”, “Biosynthesis of secondary metabolites”, “MAPK signaling pathway-plant”, and “Diterpenoid biosynthesis”. A total of 353 DEGs were identified in the comparison group of B144-T vs. B116-T, which were mainly concentrated in “Phenylpropanoid biosynthesis”, “Biosynthesis of secondary metabolites”, and “Metabolic pathways”. In contrast, only 289 and 279 DEGs were identified in B144-CK vs. B116-CK and B144-CK vs. B144-T, respectively, which is slightly less than the first two comparison groups. The main enriched pathways in B144-CK vs. B116-CK are “Biosynthesis of secondary metabolites”, “MAPK signaling pathway-plant”, and “Sesquiterpenoid and triterpenoid biosynthesis”. The DEGs of B144-CK vs. B144-T were mainly enriched in “Biosynthesis of secondary metabolites”, “Phenylpropanoid biosynthesis”, “Metabolic pathways”, and “Diterpenoid biosynthesis”. We can see from the above results that a combination of low temperature and weak light stress can significantly affect the expression of genes related to physiological processes in both materials, especially in “Biosynthesis of secondary metabolites”, “Diterpenoid biosynthesis”, and “Metabolic pathways”.

### 3.6. Differences in Antioxidant Enzyme and Gibberellin Metabolism during Compensatory Growth of Two Rice Genotypes

**Figure 10:**
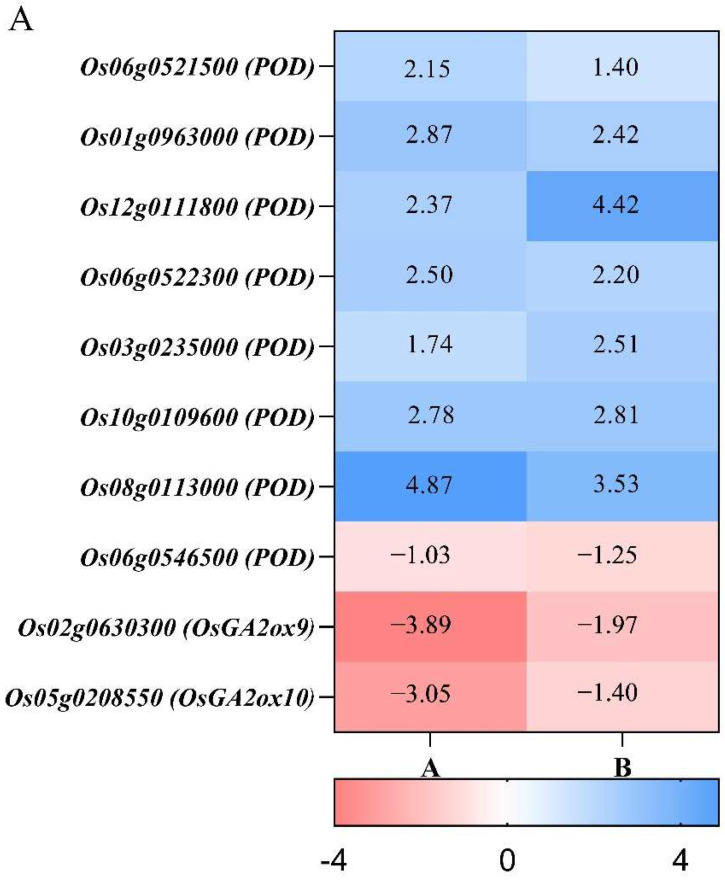

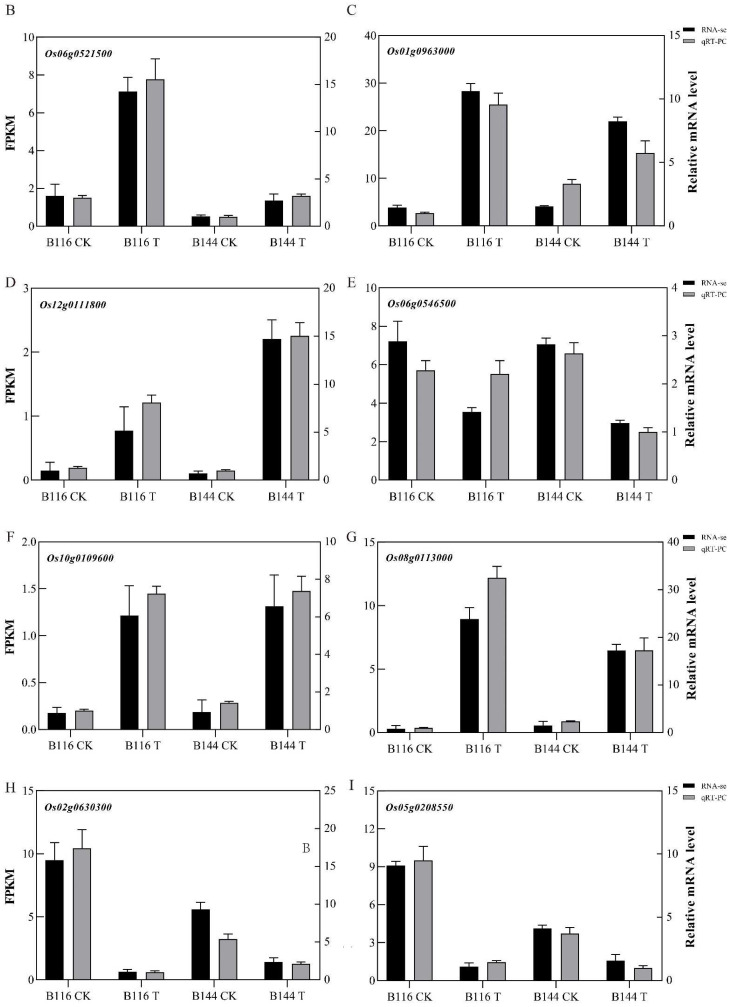
Transcript changes in peroxidase genes and gibberellin 2beta-dioxygenase genes in B116 and B144 after a combination of low temperature and weak light stress. A and B represent B116-CK vs. B116-T and B144-CK vs. B144-T, respectively (**A**). The validation of RT-PCR quantification for eight differentially expressed genes selected from Figure 10A. The left *Y*-axis and the right *Y*-axis represent the FPKM of RNA-seq and the expression level of the corresponding gene, respectively (**B**–**I**).

Comparison of the DEG analysis of different materials can help us to find the key regulatory genes for strong compensatory growth after stress. The key genes involved in the regulation of the antioxidant enzyme system and gibberellin metabolism were established by the log2(FC) analysis of co-expressed DEGs in two comparative groups, B116-CK vs. B116-T and B144-CK vs. B144-T (Figure 10 and Supplementary Table S6).

Plant antioxidant enzyme systems are closely related to abiotic stresses such as low temperature and drought. When plants are subjected to abiotic stresses, ROS accumulate rapidly in the body and cause damage to cells, while antioxidant enzymes such as SOD, POD, and CAT can scavenge excess free radicals and thus reduce the damage to plants from stress. Our study found that the transcription level of POD and the metabolism of gibberellin in rice seedlings were regulated to some extent after stress. As shown in Figure 10, a total of eight genes involved in POD coding were found and seven genes were simultaneously up-regulated after stress, while only one gene showed down-regulation. Five of the eight genes involved in encoding POD in B116-CK vs. B116-T showed higher log2(FC) compared to B144-CK vs. B144-T. Therefore, there is some difference in the regulation of genes involved in the antioxidant enzyme system between B144 and B116.

The synthesis and metabolism of gibberellin is important for plant adaptation to low temperature and rapid growth after low temperature stress. We identified two *OsGA2ox* family genes that were down-regulated in both B116-CK vs. B116-T and B144-CK vs. B144-T. This gene was also significantly more down-regulated in B116 than in B144 after stress. We selected eight genes from the above ten genes (six genes involved in POD coding and two genes involved in gibberellin metabolism) for validation via RT-PCR quantification. The results showed that their expression levels were consistent with the trend of RNA-seq, and the result further confirms our conclusion.

### 3.7. Regulation of Differentially Expressed Genes by Transcription Factors (TFs)

**Figure 11:**
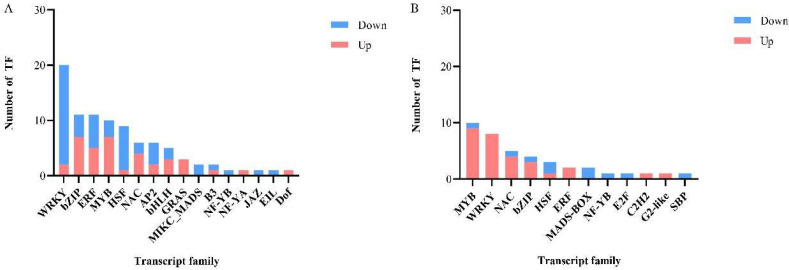
Effects of a combination of low temperature and weak light stress on the expression of transcription factors in B116 (**A**) and B144 (**B**). The *X*-axis represents the transcription factor family and the *Y*-axis represents the number of transcription factors.

Transcription factors are capable of regulating the expression of other genes and are also important in regulating plant growth and adaptation to stress. In the present experiment, we identified a total of 110 TFs from B116-CK vs. B116-T and B144-CK vs. B144-T, covering 20 TF families (Figure 11). A total of 19 TFs were universally regulated in the two comparison groups. A total of 90 TFs were identified in B116-CK vs. B116-T (much more than the 39 TFs in B144-CK vs. B144-T), in which AP2, bHLH, GRAS, B3, NF-YA, JAZ, EIL, and Dof families showed specific regulation in B116, while E2F, C2H2, G2-like, and SBP families showed specific regulation in B144. The trends in transcription factor regulation were significantly different between the two comparison groups, with B116-CK vs. B116-T showing a significantly lower number of up-regulated TFs than down-regulated, especially for the WRKY and HSF families, whereas B144-CK vs. B144-T showed the opposite trend, especially for the MYB, WRKY, NAC, and bZIP families, with only a few or no down-regulated TFs. The WRKY, bZIP, and MYB families showed relatively greater responses to stress, suggesting that they play an important role in compensatory growth after stress.

## 4. Discussion

The area of direct-seeded rice in China accounts for about 1/3 of the national rice sowing area [42], and the low temperature and weak light stress found in “late spring coldness” is extremely harmful to direct-seeded early rice production. Researchers have found a significant increase in the proportion of mild and moderate low temperatures occurring in spring in southern China and the Yangtze River Basin in recent years. [28,43]. This kind of mild and moderate “late spring coldness” is a sub-freezing stress, and the damage to rice seedlings is often non-lethal. One should not worry too much about root rot or the death of seedlings caused by this stress but should pay more attention to the compensatory growth after stress. The results showed that a combination of low temperature and weak light stress substantially reduces the height and dry matter weight of rice seedlings and retards their growth, which is similar to previous studies on the effect pattern of low temperature on rice growth [44]. At the same time, there are different performances for each material in the compensatory growth process. The traits of seedling height and dry matter weight for B116 were similar to B144 and B811 on day 0, and were significantly lower than those of the CK. However, B116 exhibited strong compensatory growth from day 0 to day 6 after stress, indicating that differences in post-stress compensatory growth ability existed among the different materials. It shows that it is not only necessary but also highly feasible to study the problem from a genotypic perspective. It has been suggested that low temperatures during the nutritional growth period can inhibit tillering in rice, which in turn reduces yield and also prolongs the total reproductive period of rice [45,46]. After the external temperature and light returned to normal, we moved the rice to the science and technology park of Jiangxi Agricultural University, Nanchang, Jiangxi province in China (28°46′ N, 115°50′ E) and investigated its yield and growth progress. The results showed that, compared to the CK, the yield of B116, B144, and B811 decreased by 8.3%, 22.9%, and 18.1%, respectively. This was mainly due to the decrease in the number of effective spikes per plant by 12.2%, 21.6%, and 25.9%, respectively. Meanwhile, the grain maturity date of B116, B144, and B811 was postponed by 3 days, 7 days, and 9 days, respectively. The extension of the growth period affected the sowing of late rice, which in turn affected the yield of late rice.

External stress can induce the overproduction of ROS, resulting in the oxidation of proteins in plants and the increased concentration of soluble proteins [47]. This process is beneficial for enhancing the tolerance of plants to stress, but it also affects the normal physiological processes of plants [48,49]. This study shows that B116 had a high soluble protein concentration on day 0 after stress but was able to rapidly decrease to levels similar to the CK during the subsequent stage, similar to the performance of the cold-tolerant variety Xiangzaoxian 6 [47]. Results of the study showed that high peroxidase activity in the low temperature and weak light stress period and a certain time after normal conditions was beneficial to the compensatory growth of rice plants. It may be the basis for its strong compensatory growth ability. In contrast, the soluble protein and H_2_O_2_ concentrations for B144 and B811 in T were always maintained at high levels, presumably due to some damage caused by stress and a severe decrease in the scavenging capacity of ROS. Therefore, the seedlings should be well protected to reduce the damage of “late spring coldness”, which will be more helpful for subsequent growth.

GS and NR activity, as catalysts for glutamine synthesis and key enzymes for nitrate assimilation, respectively, is involved in various biological reactions, such as nitrogen metabolism in plants, and has an important role in plant growth and development [45,50,51]. In this study, it was found that GS activity was relatively similar for all materials in T after stress. However, GS activity increased substantially on day 0 in B116 compared to the CK. It has been suggested that GS can regulate crops under adversity, thus improving resistance [52]. The higher activity of GS for B116 may help it to better survive the stress. NR activity was different, as NR activity for both B116 and B144 in T was substantially reduced at day 0 but was then able to increase rapidly, responding positively to compensatory growth after stress. In contrast, NR activity for B811 did not increase but showed a small decrease during the subsequent compensatory growth from stress, which might also be one of the reasons for its failure to achieve rapid compensatory growth. The recovery of carbon and nitrogen metabolism in rice can also be promoted by applying moderate amounts of nitrogen fertilizer when experiencing low temperatures in cultivation [53]. Rubisco is a key enzyme in plant carbon assimilation and photorespiration which mainly limits the photosynthetic rate by the rate of RuBP consumption, and high Rubisco activity is beneficial to the photosynthetic efficiency of rice [38,54]. Our results showed that B116 in T had no significant advantage over B144 and B811 in boosting the activity of Rubisco from 0 to 6 days. Meanwhile, no significant differences were found between B116 and B144 regarding Rubisco-related genes in the transcriptome, so we speculate that the level of photosynthetic efficiency is not a major factor in the compensatory growth for rice.

Endogenous hormones play an important synergistic role in the growth and development of plant tissues and organs, adaptation, and defense against external stresses [39,55,56]. ABA, as one of the most important stress response hormones, has various physiological effects and also plays an important role in the defense against various stresses. GA_3_, IAA, and ZR are essential plant hormones in plant growth and development and regulate processes such as seed germination, stem elongation, and fruit development. Our study showed that both B144 and B811 in T exhibited a substantial decrease in GA_3_ concentration on day 0 compared to the CK, and the decrease was consistently higher than that of B116. We also found that the changes in GA_3_ concentration for B116 and B811 in T were the exact opposite to those in the CK, indicating that B116 could better adapt to stress and that the higher GA_3_ concentration was beneficial to its stronger compensatory growth after passing the stress period. On day 0 after stress, the changes in ABA of the three total materials were not consistent in T compared to the CK. It has been reported that the ABA concentration of plants decreases gradually with increasing stress time to restore metabolism and growth, suggesting that there are some differences in the response of different genotypes in rice to low temperature and weak light stress, especially at the hormonal level [57,58]. The ABA concentration of B144 in T at day 0 after stress was much lower than the other two materials, indicating that it was less tolerant to stress and suffered large irreversible damage during stress. Therefore, despite the high GA_3_ concentration of B144, it was difficult to achieve rapid compensatory growth. In contrast to B144, B811 was able to regulate ABA concentration in a timely manner according to changes in temperature and light; however, its GA_3_ concentration was too low to achieve rapid compensatory growth. The ABA concentration of B116 in T remained at a high level from day 6 to day 12, which was somewhat inconsistent with the strong compensatory growth phenotype. However, this result is similar to the change in ABA concentration in the low temperature study of the low temperature cold-tolerant variety Xiangzaoxian 6 [22]. Low temperature tends to increase ABA concentration in rice, while decreasing GA, IAA, and ZR concentration. Higher endogenous hormone concentrations are beneficial for helping rice to survive low temperature stress and for enhancing its nitrogen utilization efficiency [59,60]. Meanwhile, the ZR and IAA concentration of B116 in T was always maintained at a higher level, especially from day 0 to day 6, which could help it to recover compensatory growth after stress.

KEGG pathway analysis of the differentially expressed genes of B116-CK and B116-T and B144-CK and B144-T showed that they were significantly enriched in “Biosynthesis of secondary metabolites”, “Diterpenoid biosynthesis”, and “Metabolic pathways”. Further analysis showed some differences in antioxidant enzyme systems and gibberellin metabolism between the two materials. In the comparison of co-expressed genes, we found that the POD-related gene log2(FC) was generally higher in B116 than in B144. Meanwhile, we identified CAT synthesis-related genes *Os02g0115700* (Supplementary Table S6) in the “Biosynthesis of secondary metabolites” pathway of B116-CK vs. B116-T, its log2(FC) was 1.41, and we did not find similar genes in B144-CK vs. B144-T. The activity of antioxidant-related enzymes in subsequent experiments needs to be further measured and verified. For gibberellin metabolism, we identified three co-expressed *OsGA2ox* family genes: *Os05g0560900* (*OsGA2ox8*), *Os02g0630300* (*OsGA2ox9*), and *Os05g0208550* (*OsGA2ox10*). This gene inactivates gibberellin via the 2β-hydroxylation pathway [56,61]. Meanwhile, *Os01g0209700* (*OsGA2ox7*) was identified only in B116-CK vs. B116-T, and its log2(FC) was −2.88. For both genes mentioned above, the degree of down-regulation in B116-CK vs. B116-T was also significantly greater than that of B144-CK vs. B144-T. These results were confirmed with each other by the previous changes in GA_3_ concentrations for B116 and B144. We identified the NR-related genes *Os08g0468100* (*OsNR1*) and *Os08g0468700* (*OsNR1.2*) in the nitrogen metabolism pathway of B116-CK vs. B116-T, which had log2(FC) of 1.636 and 2.625, respectively (Supplementary Table S6). No similar gene was found in B144-CK vs. B144-T, further validating the previous conclusion. In terms of transcription factors, we identified far more TFs from B116-CK vs. B116-T than B144-CK vs. B144-T, indicating that B116 is able to respond rapidly to environmental changes through transcription factors, which lays a good foundation for subsequent compensatory growth.

## 5. Conclusions

**Figure 12:**
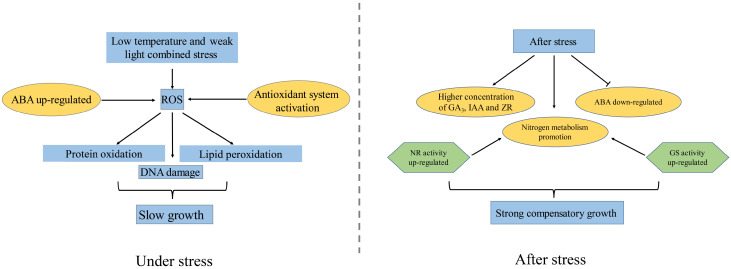
Physiological changes of strong compensatory growth materials under and after the combination of low temperature and weak light stress.

The three rice genotypes differed significantly in their ability to compensate for growth after a 4-day combined treatment of low temperature and weak light. A high concentration of ABA could reduce stress damage for the strong compensatory growth genotype of B116 during stress. Additionally, B116 has better antioxidant capacity, higher GS and NR activity, and higher GA_3_, IAA, and ZR concentration. Furthermore, it also has strong ability regarding nitrogen metabolism. All these factors may be important for its strong compensatory growth after stress. The weak compensatory growth genotype of B144 had a lower ABA concentration, weak tolerance to stress, and suffered greater irreversible damage. Therefore, even though it had strong nitrogen metabolism ability and high growth-promoting hormone levels, it was still unable to achieve rapid compensatory growth. B811 was more resistant to stress than B144, and was able to adjust ABA concentration rapidly after stress; however, its weaker nitrogen metabolism ability and lower growth-promoting hormone level made it impossible to achieve rapid compensatory growth as well. In summary, the tolerance of rice to stress and strong compensatory growth ability after stress are two different mechanisms. Rapid compensatory growth after stress requires not only a certain level of stress tolerance, but also needs a strong nitrogen metabolism and higher levels of the growth-promoting hormones GA_3_, IAA, and ZR to promote growth after stress, as summarized in Figure 12. The results of this study provide a theoretical basis for the breeding of rice with strong compensatory growth ability after a combination of low temperature and weak light stress and for the cultivation of direct-seeded early rice that can cope with “late spring coldness” in the double-seeded rice area of southern China and the Yangtze River Basin. It also has certain significance for developing crops that can deal with low temperature stress, especially in terms of rapid compensatory growth.

## Data Availability

Data available in a publicly accessible repository that does not issue DOIs. Publicly available datasets were analyzed in this study. These data can be found here: [https://www.ncbi.nlm.nih.gov/sra/PRJNA857279] (accessed on 13 July 2022).

## Supplementary Materials

The following supporting information can be downloaded at: https://www.mdpi.com/article/10.3390/plants11192523/s1, Figure S1. GO enrichment analysis of DEGs in the B144-CK vs. B116-CK comparison group, where the *X*-axis represents different GO terms and the *Y*-axis represents the number of DEGs, was grouped by biological processes, cellular components, and molecular functions based on rice GO annotation information. (A) Biological process, (B) molecular function, (C) cellular component. Figure S2. GO enrichment analysis of DEGs in the B144-T vs. B116-T comparison group, where the *X*-axis represents different GO terms and the *Y*-axis represents the number of DEGs, was grouped by biological processes, cellular components, and molecular functions based on rice GO annotation information. (A) Biological process, (B) molecular function, (C) cellular component. Figure S3. Top 20 from the KEGG pathway enrichment analysis of the DEGs. (A) and (B) represent B144-CK vs. B116-CK and B144-T vs. B116-T respectively. The *X*-axis indicates the rich factor (divide the number of differential genes in the pathway by all the numbers in the pathway) and the *Y*-axis indicates the KEGG pathway. Circle size indicates the number of DEGs in the pathway and the colors indicate the Q value of the DEGs. Table S1. List of qRT-PCR primers used. Table S2. The top 20 metabolic pathways in the B116-CK vs. B116-T comparison group. Table S3. The top 20 metabolic pathways in the B144-T vs. B116-T comparison group. Table S4. The top 20 metabolic pathways in the B144-CK vs. B116-CK comparison group. Table S5. The top 20 metabolic pathways in the B144-CK vs. B144-T comparison group. Table S6. The genes involved in “Biosynthesis of secondary metabolites”, “Metabolic pathways”, and “Nitrogen metabolism”.
