# Supplementary material for: Physiological and Transcriptomic Analyses Reveal the Mechanisms of Compensatory Growth Ability for Early Rice after Low Temperature and Weak Light Stress
Source: Plants (Basel). 2022 Sep 26;11(19):2523. doi: 10.3390/plants11192523 (PMC9570567; doi:10.3390/plants11192523)
Supplement: Supplementary file 1 [file plants-11-02523-s001.zip › plants-1920938-supplementary.pdf]

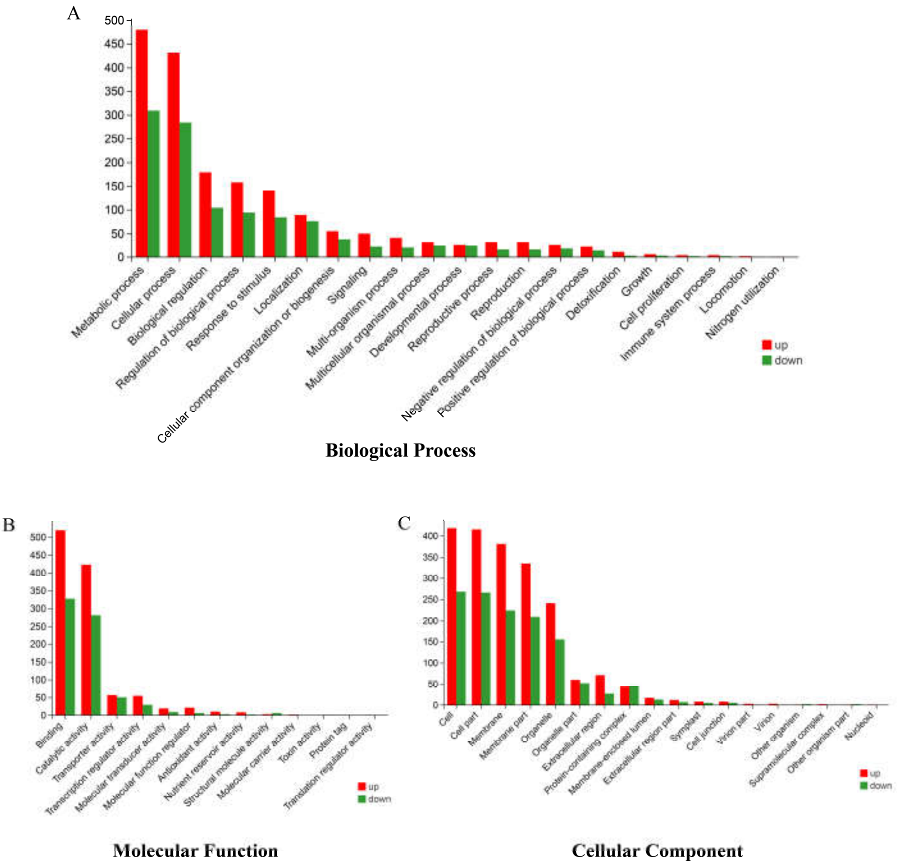

Figure S1. GO enrichment analysis of DEGs in the B144-CK vs B116-CK comparison group, where the X-axis represents different GO terms and the Y-axis represents the number of DEGs, was grouped by biological processes, cellular components and molecular functions based on rice GO annotation information. (A) Biological process, (B) Molecular function, (C) Cellular component.

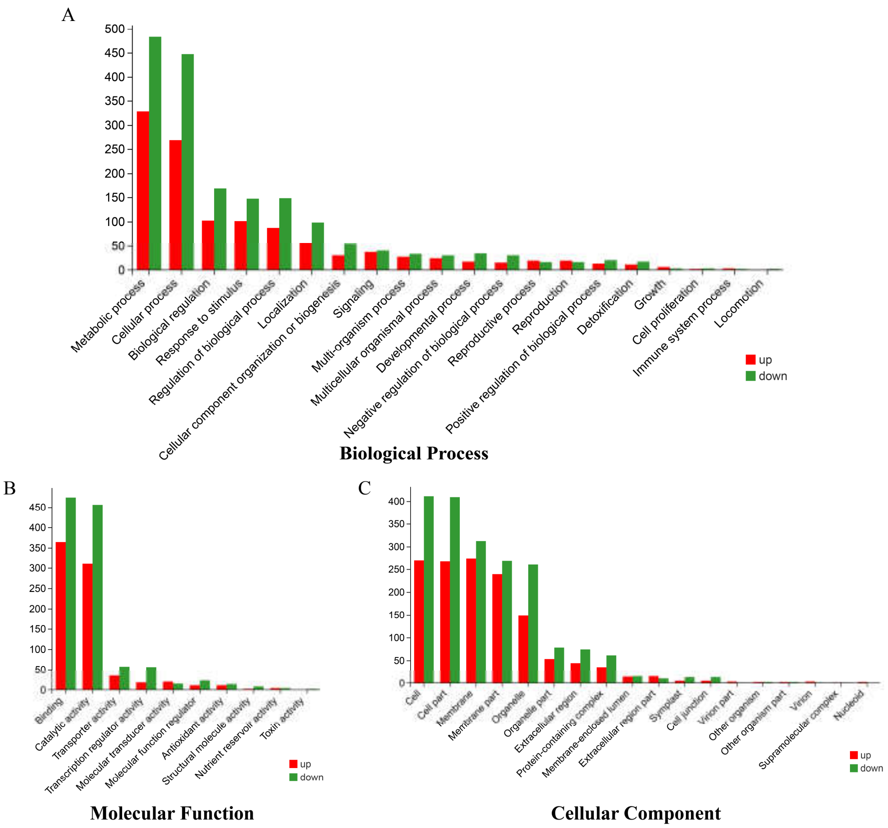

Figure S2. GO enrichment analysis of DEGs in the B144-T vs B116-T comparison group, where the X-axis represents different GO terms and the Y-axis represents the number of DEGs, was grouped by biological processes, cellular components and molecular functions based on rice GO annotation information. (A) Biological process, (B) Molecular function, (C) Cellular component.

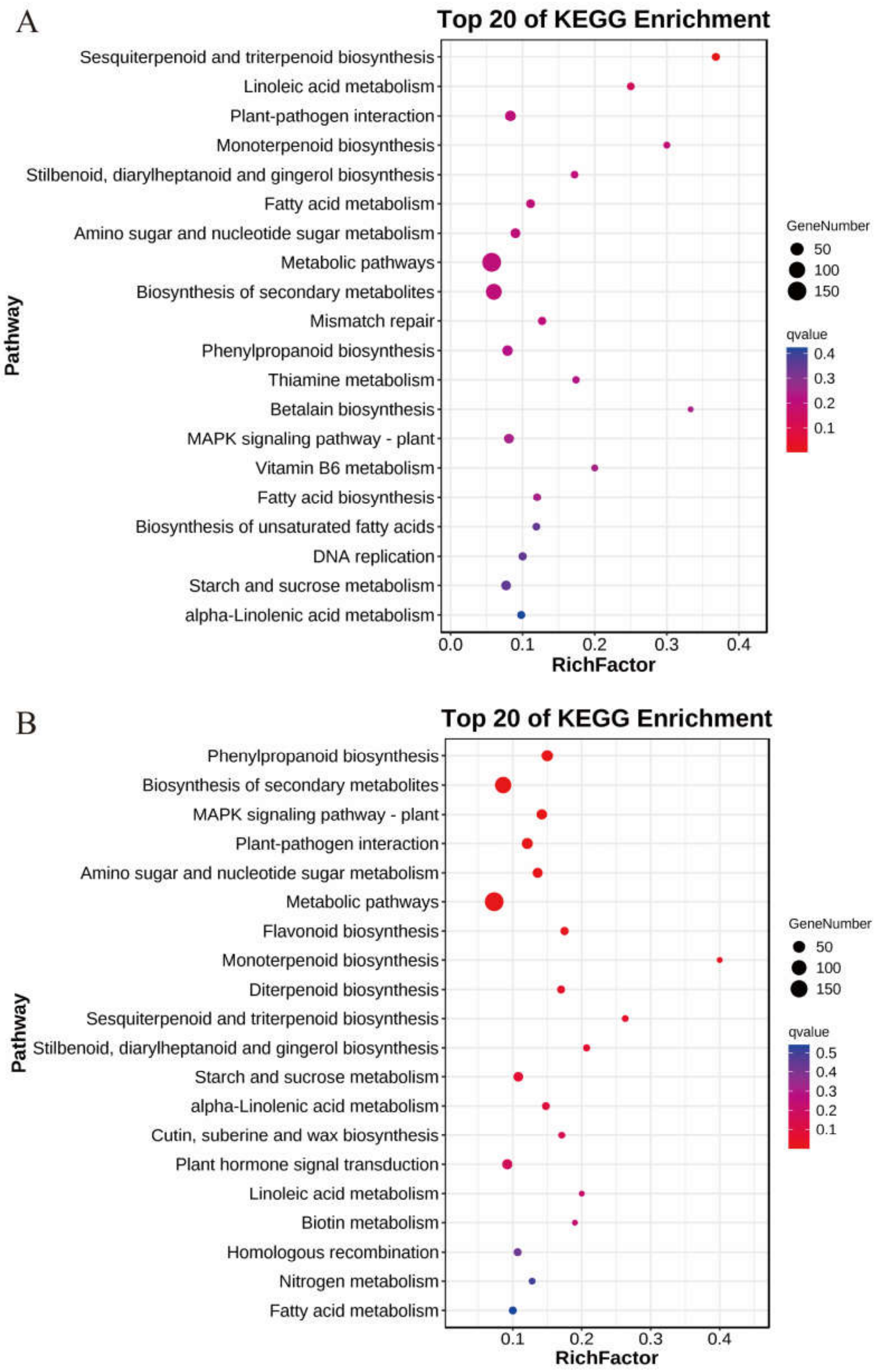

Figure S3. Top 20 of KEGG pathway enrichment analysis of the DEGs.(A),(B),represent B144-CK vs B116-CK and B144-T vs B116-T respectively. The X-axis indicates the rich

factor (Divide the number of differential genes in the pathway by all the numbers in the pathway) and the Y-axis indicates the KEGG pathway. Circle size indicates the number of DEGs in the pathway and the colors indicate Q value of DEGs.

Table S1. List of qRT-PCR primers used.

Primer	Sequence (5' to 3')	Base number
Os06g0521500-F	CGTCAATGGTTGTGATGGCTCC	24
Os06g0521500-R	GTAACGAAGCGCGAGTCCTT	22
Os01g0963000-F	GAACACCAACGACACCACCGT	25
Os01g0963000-R	GACGTACTGGTCGAAGAAGGCC	24
Os12g0111800-F	CACTCTCAGGGGCTCACACC	23
Os12g0111800-R	CACCCTTCTTGTTCAGCAGGTTC	25
Os03g0235000-F	GTCTTATCAGGGGCGCACAC	25
Os03g0235000-R	TCGGCGGAGTTGACGTCCAG	19
Os10g0109600-F	CGTCCTATCAGGAGGGCACAC	24
Os10g0109600-R	GCGGAGGTGATGTCCAGCAC	23
Os08g0113000-F	CGTCCTATCAGGAGGGCACAC	24
Os08g0113000-R	CGTAGGCGGAGGTGATGTCCA	24
Os02g0630300-F	CCTTTTTCAGGCGTGGAGCAAC	24
Os02g0630300-R	CCTGCACCCTTCGCCTGTAC	24
Os05g0208550-F	GGAGATGTCTTACAGCCAGGTGG	24
Os05g0208550-R	TGTTGGAGGGGGTAGAAGGCCC	24
OsActin-F	GGCCAACAGGGAGAAGATGACAC	23
OsActin-R	GATCCCTACCAGCAAGATCAAGACG	25

Table S2. The top 20 metabolic pathways in B116-CK vs B116-T comparable group.

Top 20 metabolic pathway	Number of DEGs	Q value
MAPK signaling pathway - plant	45	0
Plant hormone signal transduction	52	0
Diterpenoid biosynthesis	18	0
Plant-pathogen interaction	47	0
Linoleic acid metabolism	9	0.000043
Biosynthesis of secondary metabolites	140	0.000219
Phenylpropanoid biosynthesis	33	0.005342
alpha-Linolenic acid metabolism	12	0.007533
Tryptophan metabolism	13	0.025788
Flavonoid biosynthesis	11	0.027755
Benzoxazinoid biosynthesis	2	0.116982
Metabolic pathways	203	0.128875
Monoterpenoid biosynthesis	3	0.193994
Cyanoamino acid metabolism	9	0.220487
Arginine and proline metabolism	9	0.245645
Stilbenoid, diarylheptanoid and gingerol biosynthesis	5	0.245645
Nitrogen metabolism	6	0.245645
Glycerophospholipid metabolism	14	0.245645
Glycerolipid metabolism	11	0.28108
Betalain biosynthesis	2	0.28108

Table S3. The top 20 metabolic pathways in B144-T-vs-B116-T comparable group.

Top 20 metabolic pathway	Number of DEGs	Q_value
Phenylpropanoid biosynthesis	40	0.000007
Biosynthesis of secondary metabolites	132	0.000162
MAPK signaling pathway - plant	30	0.000361
Plant-pathogen interaction	35	0.001823
Amino sugar and nucleotide sugar metabolism	24	0.003678
Metabolic pathways	198	0.00417
Flavonoid biosynthesis	11	0.020868
Monoterpenoid biosynthesis	4	0.02987
Diterpenoid biosynthesis	9	0.052578
Sesquiterpenoid and triterpenoid biosynthesis	5	0.052578
Stilbenoid, diarylheptanoid and gingerol biosynthesis	6	0.069871
Starch and sucrose metabolism	21	0.069871
alpha-Linolenic acid metabolism	9	0.094355
Cutin, suberine and wax biosynthesis	6	0.142625
Plant hormone signal transduction	26	0.175656
Linoleic acid metabolism	4	0.212377
Biotin metabolism	4	0.235401
Homologous recombination	9	0.428571
Nitrogen metabolism	5	0.508869
Fatty acid metabolism	9	0.541201

Table S4. The top 20 metabolic pathways in B144-CK-vs-B116-CK comparable group.

Top 20 metabolic pathway	Number of DEGs	Q value
Sesquiterpenoid and triterpenoid biosynthesis	7	0.00232
Linoleic acid metabolism	5	0.134145
Plant-pathogen interaction	24	0.200238
Monoterpenoid biosynthesis	3	0.200238
Stilbenoid, diarylheptanoid and gingerol biosynthesis	5	0.200238
Fatty acid metabolism	10	0.200238
Amino sugar and nucleotide sugar metabolism	16	0.200238
Metabolic pathways	154	0.200238
Biosynthesis of secondary metabolites	93	0.200238
Mismatch repair	7	0.200238
Phenylpropanoid biosynthesis	21	0.227218
Thiamine metabolism	4	0.227218
Betalain biosynthesis	2	0.247306
MAPK signaling pathway - plant	17	0.247306
Vitamin B6 metabolism	3	0.247306
Fatty acid biosynthesis	6	0.247306
Biosynthesis of unsaturated fatty acids	5	0.350889
DNA replication	7	0.350889
Starch and sucrose metabolism	15	0.351344
alpha-Linolenic acid metabolism	6	0.423398

Table S5. The top 20 metabolic pathways in B144-CK-vs-B144-T comparable group.

Top 20 metabolic pathway	Number of DEGs	Q_value
Biosynthesis of secondary metabolites	134	0
Phenylpropanoid biosynthesis	41	0
Diterpenoid biosynthesis	18	0
Metabolic pathways	182	0
Amino sugar and nucleotide sugar metabolism	24	0.000066
Starch and sucrose metabolism	23	0.000884
Glutathione metabolism	16	0.007766
Cyanoamino acid metabolism	10	0.012728
MAPK signaling pathway - plant	21	0.012728
Flavonoid biosynthesis	8	0.093888
Tyrosine metabolism	7	0.093888
Sesquiterpenoid and triterpenoid biosynthesis	4	0.094416
Biosynthesis of unsaturated fatty acids	6	0.109163
Betalain biosynthesis	2	0.214
Indole alkaloid biosynthesis	1	0.298129
Isoquinoline alkaloid biosynthesis	4	0.298129
Fatty acid metabolism	8	0.389815
Monoterpenoid biosynthesis	2	0.41668
Glucosinolate biosynthesis	2	0.41668
Cutin, suberine and wax biosynthesis	4	0.421257

Table S6. The genes involved in "Biosynthesis of secondary metabolites", "Metabolic pathways", and "Nitrogen metabolism".

Gene id		B116-CK vs B116-T log2(FC)	B144-CK vs B144-T log2(FC)	Description
POD	*Os06g0521500*	2.145	1.399	peroxidase
POD	*Os01g0963000*	2.873	2.423	peroxidase
POD	*Os12g0111800*	2.366	4.416	peroxidase
POD	*Os06g0546500*	-1.028	-1.246	peroxidase
POD	*Os06g0522300*	2.497	2.196	peroxidase
POD	*Os03g0235000*	1.742	2.507	peroxidase
POD	*Os10g0109600*	2.780	2.815	peroxidase
POD	*Os08g0113000*	4.867	3.531	peroxidase
OsGA2ox8	*Os05g0560900*	-3.723	-1.589	gibberellin 2beta-dioxygenase
OsGA2ox9	*Os02g0630300*	-3.891	-1.975	gibberellin 2beta-dioxygenase
OsGA2ox10	*Os05g0208550*	-3.048	-1.397	gibberellin 2beta-dioxygenase
CAT	*Os02g0115700*	1.410	NA	catalase
OsGA2ox7	Os01g0209700	-2.877	NA	gibberellin 2beta-dioxygenase
OsNR1	*Os08g0468100*	1.636	NA	Nitrate reductase
OsNR1.2	*Os08g0468700*	2.625	NA	Nitrate reductase
